# A Polyphenolic Extract from Olive Mill Wastewaters Encapsulated in Whey Protein and Maltodextrin Exerts Antioxidant Activity in Endothelial Cells

**DOI:** 10.3390/antiox8080280

**Published:** 2019-08-05

**Authors:** Konstantina Kreatsouli, Zinovia Fousteri, Konstantinos Zampakas, Efthalia Kerasioti, Aristidis S. Veskoukis, Christos Mantas, Paschalis Gkoutsidis, Dimitrios Ladas, Konstantinos Petrotos, Demetrios Kouretas, Dimitrios Stagos

**Affiliations:** 1 Department of Biochemistry and Biotechnology, University of Thessaly, Viopolis, 41500 Larissa, Greece; 2 Department of Biosystem Engineering, Technical Education Institute of Thessaly, 41110 Larissa, Greece

**Keywords:** olive mill wastewater, encapsulation, maltodextrin, whey protein, gelatin, spray drying, antioxidants, glutathione, endothelial cells

## Abstract

The aim of the present study was to compare maltodextrin and whey protein as encapsulation carriers for olive mill wastewater (OMWW) phenolic extract for producing antioxidant powder, by using spray drying under 17 different conditions. In some samples, gelatin was also added in the encapsulation mixture. The antioxidant activity was assessed in vitro by using the DPPH^•^, ABTS^•+^, reducing power and DNA plasmid strand breakage assays. The results showed that both materials were equally effective for producing antioxidant powder, although by using different conditions. For example, inlet/outlet temperature of the spray drying did not seem to affect the maltodextrin samples’ antioxidant activity, but whey protein samples showed better antioxidant activity at lower temperatures. Gelatin use decreased antioxidant activity, especially in whey protein samples. The two most potent samples, one encapsulated in maltodextrin and the other in whey protein, were examined for their antioxidant effects in human endothelial cells by assessing glutathione (GSH) and reactive oxygen species (ROS) levels. Both samples significantly enhanced the antioxidant molecule of GSH, while maltodextrin sample also decreased ROS. The present findings suggested both materials for encapsulation of OMWW extract for producing antioxidant powder which may be used in food products, especially for the protection from ROS-induced endothelium pathologies.

## 1. Introduction

Olive mill wastewaters (OMWWs) are byproducts of the olive oil production process, causing significant problems such as soil contamination and eutrophication, when they are discarded in the environment. Although OMWWs have been extensively considered as a byproduct, several studies have shown that they are rich in polyphenolic compounds (e.g., oleuropein, tyrosol, hydroxytyrosol, coumaric acid, caffeic acid, vanillic acid, ferulic acid, kaempferol, and quercetin) with important biological activities [1]. For example, phenolic compounds are widely known for their antioxidant properties [2]. Our research group has previously demonstrated that administration of feed supplemented with polyphenols from OMWW improves the redox status in farm animals [3,4,5].

Polyphenolic extracts from OMWW may be used as food supplements or preservatives, but their unpleasant bitter taste is a significant problem. Furthermore, the polyphenolic compounds can be chemically unstable under the influence of temperature, light, and oxygen [6]. Therefore, the encapsulation of polyphenolic extracts is an effective way to preserve their stability and bioactivity, while overcoming the problem of bad taste and simultaneously enhancing their bioavailability [7]. Spray drying encapsulation is a common and popular technique in terms of production cost, short process time, and low thermal stresses [8].

The entire vascular system consists of a monolayer of endothelial cells, the integrity of which is necessary to maintain a harmonic circulatory function. Apart from that, the endothelium regulates the homeostasis as well as the immune and inflammatory responses of the body [9,10]. Oxidative stress can severely damage the endothelium; thus, it constitutes one of the most important factors of pathological conditions of the vessels, along with atherosclerosis and thrombosis [10].

Thus, the aim of the present study was initially to evaluate the antioxidant potency of a polyphenolic extract from OMWW, encapsulated in three different carrier agents (i.e., maltodextrin, whey protein and gelatin) under different conditions, thus producing 17 samples. Then, the two samples exhibiting the greatest antioxidant activity (i.e., 3 and 9) were chosen in order to assess their antioxidant effects on the redox status of human endothelial cells.

## 2. Materials and Methods

### 2.1. Preparation of Encapsulation Powders

The liquid raw material that was used for the production of the OMWW originating antioxidant powders was supplied by Polyhealth S.A. (Larissa, Greece). This liquid raw material was a standard product of Polyhealth S.A., commercialized under the trademark ‘MEDOLIVA^®^ Liquid’. The polyphenolic composition of MEDOLIVA^®^ liquid as assessed by HPLC analysis has been previously reported [11]. Thus, a mixed liquid material was prepared by mixing MEDOLIVA^®^ liquid (an aqueous solution) with Twin 80 (liquid surfactant) and with either plain or mixed encapsulation agents (i.e., whey protein C80, maize maltodextrin DE18, and gelatin) according to the recipes given in detail in Table 1. Consequently, the mixture was thoroughly homogenized by ultrasonic energy. Finally, the homogenized aqueous solution was spray dried for water removal and production of 17 dry encapsulated OMWW polyphenol powders. A BUCHI Spray Dryer model B-290 connected with a B296 Dehumidifier was used to carry out the spray drying. The operating conditions used for each recipe are presented in Table 1. The conditions and the ingredient ratios given in Table 1 were selected after more than 100 trials (data not shown) and only 17 were found to be acceptable on the basis of giving good quality and stable powder at a yield of more than 75% of the initial aqueous solution dry matter. The initial temperature range tested was from 100 to 220 °C, while the gelatin percentage tested was from 5 to 30 *w*/*w*% of the maltodextrin or whey protein. In many cases, the tested conditions led to a failure to obtain powder and the finished product was slurry type or very sticky due to unfavorable glass transition conditions.

### 2.2. Free-Radical Scavenging Activity

The free-radical scavenging activity of the powders was evaluated using the 2,2’-azino-bis (3-ethylbenzthiazoline-6-sulfonic acid) (ABTS^•+^) and 2,2-diphenyl-picrylhydrazyl (DPPH^•^) radical scavenging assays as previously described [12].

In the DPPH assay, 950 μL of 100 μM methanolic solution of DPPH^•^ was mixed with 50 μL of the tested powder at different concentrations. Regarding the DPPH^•^ assay, 1.0 mL of freshly made methanolic solution of DPPH^•^ radical (100 μM) was mixed with the powder solution at different concentrations. The contents were vigorously mixed, incubated at room temperature in the dark for 20 min and the absorbance was measured at 517 nm. The measurement was conducted on a Hitachi U-1900 ratio beam spectrophotometer (Tokyo, Japan). In each experiment, the tested powder alone in methanol was used as blank and DPPH^•^ alone in methanol was used as control.

In the ABTS^•+^ assay, ABTS^•+^ radical was produced by mixing 2 mM ABTS with 30 μM H_2_O_2_ and 6 μM horseradish peroxidase (HRP) enzyme in 1 mL of distilled water. The solution was vigorously mixed and incubated at room temperature in the dark for 45 min until ABTS^•+^ radical formation. Then, 10 μL of different powder concentrations were added in the reaction mixture and the absorbance at 730 nm was read. The measurement was conducted on a Hitachi U-1900 ratio beam spectrophotometer (Tokyo, Japan). In each experiment, the tested powder in distilled water containing ABTS and H_2_O_2_ was used as blank, and the ABTS^•+^ radical solution with 10 μL H_2_O was used as control.

The percentage of the radical scavenging capacity (RSC) of the tested powders for both assays was calculated according to the following equation:

Radical scavenging capacity (%) = [(A_control_ − A_sample_)/A_control_] × 100 (1)

where, A_control_ and As_ample_ are the absorbance values of the control and the tested samples, respectively. Moreover, in order to compare the radical scavenging capacity of the samples, the IC_50_ value showing the concentration that induced 50% scavenging of DPPH^•^ and ABTS^•+^ was calculated. In both assays vitamin C was used as a positive control. All experiments were carried out in triplicate and at least on two separate occasions.

### 2.3. Peroxyl-Radical-Induced Plasmid DNA Strand Cleavage

The peroxyl-radical-induced DNA plasmid strand cleavage assay was performed as described previously [12]. In brief, peroxyl radicals (ROO) were produced from thermal decomposition of 2,2′-azobis(2-amidinopropane hydrochloride) (AAPH). The reaction mixture (10 μL) containing 1 μg Bluescript-SK+ plasmid DNA, 2.5 mM AAPH in phosphate-buffered saline (PBS) and the tested powder at different concentrations was incubated in the dark for 45 min at 37 °C. Then, the reaction was stopped by the addition of 3 μL loading buffer (0.25% bromophenol blue and 30% glycerol). After analyzing the DNA samples by agarose gel electrophoresis, they were photographed and analyzed using the Alpha Innotech Multi Image (ProteinSimple, San Jose, CA, USA). In addition, plasmid DNA was treated with each powder alone at the highest concentration used in the assay in order to test their effects on plasmid DNA conformation. The percentage of the protective activity of the tested powders from ROO-induced DNA strand breakage was calculated using the following equation:

% Inhibition = [(S − S_o_)/(S_control_ − S_o_)] × 100 (2)

where, S_control_ is the percentage of supercoiled DNA in the negative control (plasmid DNA alone), S_o_ is the percentage of supercoiled plasmid DNA in the positive control (without the tested powder but in the presence of the radical initiating factor), and S is the percentage of supercoiled plasmid DNA in the tested powder along with the radical initiating factor. Moreover, the IC_50_ values showing the concentration that inhibited the AAPH-induced DNA relaxation by 50% were calculated. Vitamin C was used as a positive control. At least three independent experiments were performed for each sample.

### 2.4. Reducing Power

Reducing power was determined spectrophotometrically as described previously [13]. _The RP_0.5AU_ value showing the powder concentration that caused an absorbance of 0.5 at 700 nm was calculated from the graph plotting absorbance against powder concentration. Vitamin C was used as a positive control. At least two independent experiments in triplicate were performed for each tested sample.

### 2.5. Evaluation of Relative Antioxidant Capacity Index (RACI)

In order to find out which samples exhibited the highest antioxidant activity in all antioxidant assays, the RACI was evaluated for each sample as described in Sun and Tanumihardjo [13]. RACI is the mean value of standard scores evaluated by initial data generated with different methods for a sample. A standard score was calculated according to the following equation:

Standard score = (x − μ)/σ(3)

where x was the raw data, μ was the mean of all samples’ values of each method, and σ was the standard deviation.

Since in all antioxidant assays the raw data were IC_50_ values, the lower the RACI value the higher the antioxidant capacity.

### 2.6. Cell Culture Conditions

As previously described [14], human endothelial EA.hy926 cells gifted from Prof. Koukoulis (University of Thessaly, Greece) were cultured in normal Dulbecco’s modified Eagle’s medium (DMEM) in plastic disposable tissue culture flasks at 37 °C in 5% carbon dioxide.

### 2.7. XTT Cell Viability Assay

The antioxidant activity of the powders in EA.hy926 cells was examined using non-cytotoxic concentrations. In order to select these concentrations, the cytotoxicity of the powders was checked using the XTT cell viability assay kit (Sigma) as previously described [14]. Briefly, EA.hy926 cells were seeded into a 96-well plate (1 × 10^4^ cells per well) in DMEM containing 10% fetal bovine serum FBS. After 24 h incubation at 37 °C in 5% CO_2_, the cells were treated with different concentrations of the powders in FBS-free DMEM and incubated for another 24 h. Then, 50 μL of XTT test solution was added to each well. After 4 h of incubation, absorbance was measured at 450 nm and also at 630 nm as a reference wavelength in a Bio-Tek ELx800 microplate reader (Winooski, VT, USA). Negative control was DMEM serum-free medium. The absorbance values of the control and powders were used for calculating the percentage inhibition of cell growth caused by the powder treatment. All experiments were carried out in triplicate and on two separate occasions.

### 2.8. Treatment of EA.hy926 Cells with the Powders

The cells were cultured until 75%–80% confluence of the flask. Afterwards the medium was replaced with serum-free medium containing the tested powders at non-cytotoxic concentrations. The cells were treated with the powders for 24 h, and then they were trypsinized, collected, and centrifuged twice at 300× *g* for 10 min at 5 °C. At the end of the first centrifugation, the supernatant fluid was discarded and the cellular pellet was resuspended in PBS. After the second centrifugation, the cell pellet was collected and used for measuring the glutathione (GSH) and reactive oxygen species (ROS) levels.

### 2.9. Assessment of GSH and ROS Levels in EA.hy926 Cells by Flow Cytometry

The GSH and ROS levels in EA.hy926 cells were assessed using mercury orange and 2,7-dichlorofluorescein diacetate (DCF-DA), respectively, as described previously [9]. In brief, the cells were re-suspended in PBS (10^6^ cells/mL) and incubated in the presence of mercury orange (10 μΜ) or DCF-DA (40 μΜ) respectively, in the dark at 37° C for 30 min. Then, the cells were washed, re-suspended in PBS, and subjected to flow cytometric analysis using a FACSCalibur flow cytometer (Becton Dickinson, Franklin Lakes, NJ, USA) with excitation and emission wavelengths at 488 and 530 nm for ROS, and at 488 and 580 nm for GSH. Data were analyzed using ‘BD Cell Quest’ software (Becton Dickinson). Each experiment was repeated at least three times.

### 2.10. Statistical Analysis

All results were expressed as mean ± standard deviation (SD). Differences were considered significant at *p* < 0.05. One-way ANOVA was performed followed by Tukey’s test for multiple pair-wise comparisons using the SPSS 20.0 software (SPSS, Inc., Chicago, IL, USA).

## 3. Results

### 3.1. Free-Radical Scavenging Activity of the Powders

In the present study, 17 powders produced by encapsulation of an OMWW polyphenolic extract by spray drying under different conditions in whey protein, maltodextrin, and gelatin were tested for their free-radical scavenging activity against DPPH^•^ and ABTS^•+^ radicals. All of them were able to scavenge DPPH^•^ with IC_50_ values ranging from 295 ± 18 to 660 ± 73 μg/mL (Table 2). Moreover, all the extracts demonstrated a potent ABTS^•+^ scavenging capacity with IC_50_ values ranging from 290 ± 9 to 710 ± 68 μg/mL (Table 2).

### 3.2. Protection from ROO^•^-Induced Plasmid DNA Strand Cleavage

All the powders protected from ROO-induced DNA damage with IC_50_ values ranging from 520 ± 41 to 2300 ± 227 μg/mL (Table 2).

### 3.3. Reducing Capacity of the Extracts

The estimation of the extracts’ reducing capacity was based on the reducing power assay by determining the RP_0.5AU_ values, which ranged from 434 ± 30 to 890 ± 18 μg/mL (Table 2). Samples 9, 12, 13, 15, 16, 17, 18, and 19 did not show absorbance of 0.5 at 700 nm at the tested concentrations (Table 2).

### 3.4. Determination of Extracts’ Non-Cytotoxic Concentrations in EA.hy926 Cells

**Figure 1:**
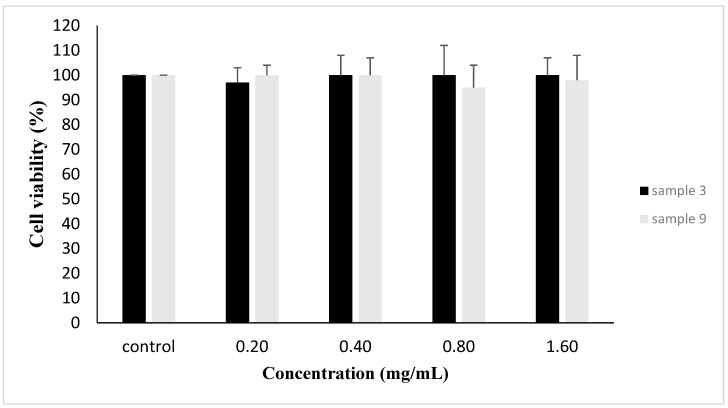
Cell viability following the treatment of EAhy.926 cells with samples 3 and 9. The results are presented as the means ± SEM of three independent experiments carried out in triplicate. * *p* < 0.05 indicates significant difference from the control value.

Based on the results from DPPH^•^, ABTS^•+^, ROO-induced DNA damage, and reducing power, the two most potent of the powders were selected in order to examine their antioxidants effects in endothelial EAhy.926 cells. Specifically, the selection was made according to the RACI value that each powder had in all the above assays (Table 2). Since the RACI values were based on the IC_50_ values, the lower the RACI value the higher the antioxidant capacity. Thus, the two most potent samples were 3 and 9. However, before examining the powders’ antioxidant effects in EAhy.926 cells, their cytotoxicity was assessed using the XTT assay in order to select non-cytotoxic concentrations. The results showed that both powders had no significant cytotoxicity at concentrations up to 1600 μg/mL (Figure 1).

### 3.5. Effects of Powders on GSH Levels in EA.hy926 Cells

**Figure 2:**
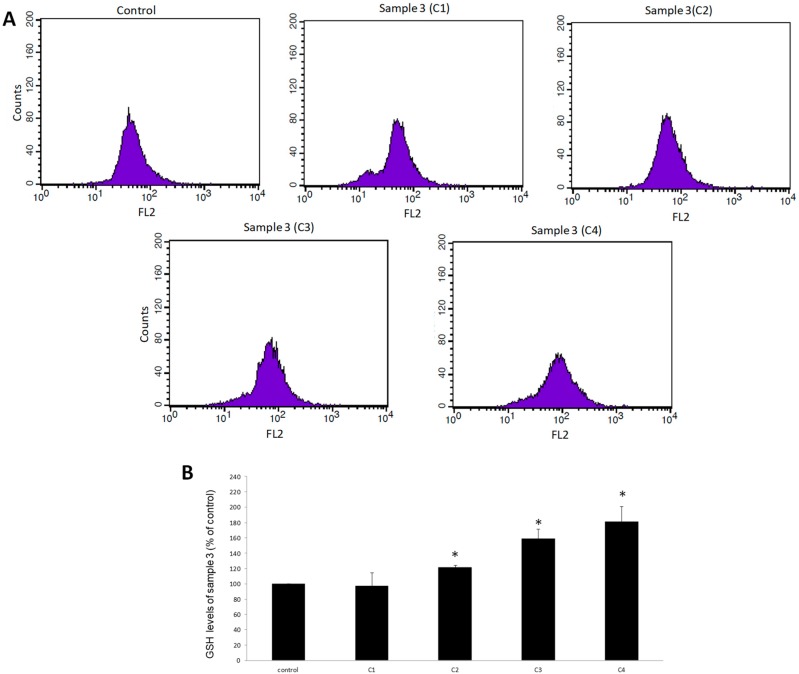
The effects of sample 3 on glutathione (GSH) levels in EA.hy926 cells after treatment for 24 h, as assessed by flow cytometry. (**A**) The histograms of cell counts versus fluorescence of 10,000 cells after treatment with sample 3. (**B**) Bar charts demonstrate the GSH levels as % of control as estimated by the histograms in EA.hy926 cells after treatment. C1, C2, C3, C4: 200, 400, 800, and 1600 μg/mL, respectively. * Statistically significant compared to the control cells. FL2: The detection of fluorescence using 488 and 580 nm as the excitation and emission wavelength, respectively.

**Figure 3:**
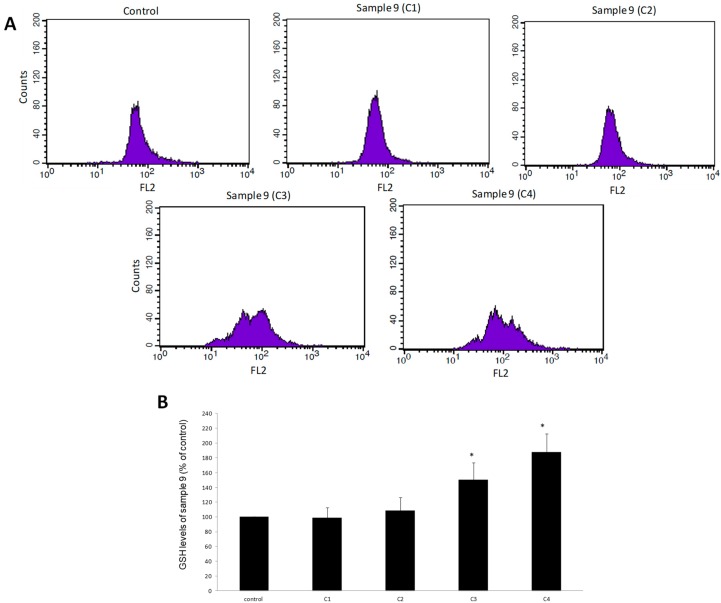
The effects of sample 9 on GSH levels in EA.hy926 cells after treatment for 24 h, as assessed by flow cytometry. (**A**) The histograms of cell counts versus fluorescence of 10,000 cells after treatment with sample 9. (**B**) Bar charts demonstrate the GSH levels as % of control as estimated by the histograms in EA.hy926 cells after treatment. C1, C2, C3, C4: 200, 400, 800, and 1600 μg/mL, respectively. * Statistically significant compared to the control cells. FL2: The detection of fluorescence using 488 and 580 nm as the excitation and emission wavelength, respectively.

For assessing the effects of samples 3 and 9 on GSH levels in EA.hy926 cells, flow cytometry analysis was used. The results showed that treatment of EA.hy926 cells with sample 3 significantly increased GSH levels by 22%, 59%, and 82% at 400, 800, and 1600 μg/mL, respectively compared to control (Figure 2). When cells were treated with sample 9, GSH levels were significantly increased by 51% and 88% at concentrations of 800 and 1600 μg/mL, respectively, compared to the control (Figure 3).

### 3.6. Effects of Powders on ROS Levels in EA.hy926 Cells

**Figure 4:**
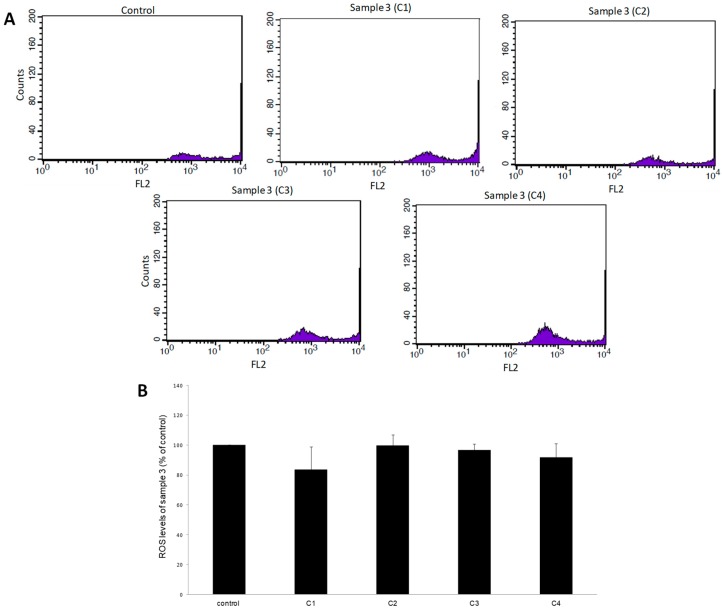
The effects of sample 3 on reactive oxygen species (ROS) levels in EA.hy926 cells after treatment for 24 h, as assessed by flow cytometry. (**A**) The histograms of cell counts versus fluorescence of 10,000 cells after treatment with sample 3. (**B**) Bar charts demonstrate the ROS levels as % of control as estimated by the histograms in EA.hy926 cells after treatment. C1, C2, C3, C4: 200, 400, 800, and 1600 μg/mL, respectively. * Statistically significant compared to the control cells. FL2: The detection of fluorescence using 488 and 580 nm as the excitation and emission wavelength, respectively.

**Figure 5:**
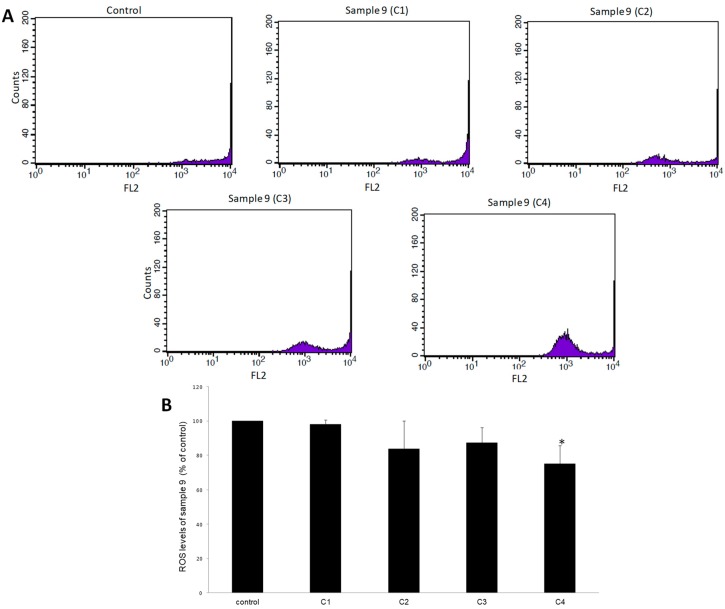
The effects of sample 9 on ROS levels in EA.hy926 cells after treatment for 24 h, as assessed by flow cytometry. (**A**) The histograms of cell counts versus fluorescence of 10,000 cells after treatment with sample 9. (**B**) Bar charts demonstrate the ROS levels as % of control as estimated by the histograms in EA.hy926 cells after treatment. C1, C2, C3, C4: 200, 400, 800, and 1600 μg/mL, respectively. * Statistically significant compared to the control cells. FL2: The detection of fluorescence using 488 and 580 nm as the excitation and emission wavelength, respectively.

Like GSH, the levels of ROS in EAhy.926 cells after treatment with samples 3 and 9 were evaluated by flow cytometry analysis. The results demonstrated that after treatment of cells with sample 3, ROS levels did not change at any concentration compared to control (Figure 4). Moreover, after cell treatment with sample 9, ROS were decreased by 25% at 1600 μg/mL, compared to control (Figure 5).

## 4. Discussion

The composition and the amounts of OMWW cause serious environmental problems in areas of olive oil production, especially when they are discharged without any previous treatment [15]. On the other hand, OMWWs contain high quantities of polyphenols with important bioactivities such as antioxidant property [1,11]. Thus, polyphenolic extracts from OMWW can be used as natural alternatives to commercial synthetic antioxidants with applications in the food industry and in the development of nutraceutical products [3,4,11,16].

However, when polyphenols are added in foods, there are problems regarding bad or bitter taste and discoloration. The encapsulation of polyphenolic extracts has been suggested as a method to overcome these problems as well as to improve polyphenols’ stability, half-life, and bioavailability [10,17]. Thus, the aim of this study was to evaluate the effects on the redox status of endothelial cells of an OMWW extract encapsulated under different conditions and encapsulation carriers using spray drying. Apart from the encapsulation carrier, the spray drying conditions of the different samples differed in the inlet and outlet temperature, percentage of pump function, and use of Tween 80. Spray drying is a process widely used for encapsulation of oils and flavors [18]. Some of the advantages of this procedure are the generation of sample powders with good quality, low water activity, and easier handling and storage, while it also protects the active material against undesirable reactions [19]. The encapsulation carriers used were maltodextrin, maltodextrin/gelatin (5:1), whey protein, and whey protein/gelatin (5:1). Maltodextrin, a hydrolyzed starch, offers advantages for encapsulation such as relatively low cost, neutral aroma and taste, low viscosity at high solid concentrations, and protection against oxidation [20]. However, the most serious drawback of this material is its low emulsifying capacity; thus, it is desirable to use it in combination with other surface-active biopolymers such as gelatin [21]. Whey proteins, like other milk proteins, are hydrocolloids exhibiting good solubility and behavior and are used over the last years in food industry due to the increasingly need for natural products [22]. There have so far been only a few studies on the encapsulation of polyphenols from OMWW. For example, it has been reported that OMWW polyphenols encapsulated in maltodextrin and maltodextrin/acacia fiber (1:1) using spray drying exhibited antioxidant and antiglycative activities as well as inhibited Maillard reaction in milk [23,24,25]. Moreover, Caporaso et al. [22] have produced antioxidant powder by encapsulating OMWW polyphenols in whey protein/xanthan gum.

At first, in vitro antioxidant assays were applied in order to select the most potent samples that were used for the examination in endothelial cells. The findings showed that although all the 17 samples exhibited free-radical scavenging activity in DPPH^•^ and ABTS^•+^ assays, there was a great variation in their effect up to about two- and threefold, respectively. In ABTS^•+^ assay, the samples encapsulated in whey protein were more potent at lower inlet/outlet temperature than at higher ones. However, inlet/outlet temperature did not seem to affect the potency of samples encapsulated in maltodextrin. Moreover, the scavenging activity against ABTS^•+^ radical was not dependent on the encapsulation carrier. For example, in the ABTS^•+^ assay, the first (No. 9) and third (No. 2) most potent samples were encapsulated in maltodextrin and whey protein, respectively. Finally, in the ABTS^•+^ assay, the use of gelatin did not significantly affect the antioxidant potency of samples encapsulated either in whey protein or maltodextrin. Unlike the ABTS^•+^ assay, in the DPPH^•^ assay, inlet/outlet temperatures had no effect on the potency of samples encapsulated in whey protein. Moreover, like the ABTS^•+^ assay, these temperatures did not seem to affect scavenging activity against DPPH^•^ of samples encapsulated in maltodextrin. Furthermore, in the DPPH^•^ assay, all the samples encapsulated in whey protein exhibited higher antioxidant activity than the samples encapsulated in maltodextrin (except No. 9). In the DPPH^•^ assay, the addition of gelatin decreased antioxidant activity in samples encapsulated in whey protein, while it did not affect the activity of samples encapsulated in maltodextrin. Moreover, some samples (e.g., No. 9) exhibited high potency in both DPPH^•^ and ABTS^•+^ assay, while other samples (e.g., No. 5) had high potency in one assay and low in the other. The observed differences between DPPH^•^ and ABTS^•+^ may be explained by the different solvents used in these assays, that is, methanol and water, respectively. Thus, lipophilic compounds are more active in the DPPH^•^ assay, while hydrophilic compounds are more active in the ABTS^•+^ assay.

Apart from the free-radical scavenging activity, the samples’ reducing capacity was assessed using the reducing power assay, since many antioxidants act as hydrogen donors. Again, the reducing capacity varied up to twofold between the samples exhibiting the higher and the lower activity. The type of encapsulation carrier did not affect reducing activity, since samples of both whey protein (e.g., No. 3) and maltodextrin (e.g., No. 9) demonstrated high potency. Moreover, reducing activity did not seem to depend on inlet/outlet temperatures in samples encapsulated either in whey protein or maltodextrin. It was also remarkable that the addition of gelatin in the encapsulation mixture decreased reducing activity in both whey protein and maltodextrin samples.

Furthermore, all the samples protected from ROS-induced DNA damage, but, like the other assays, the IC_50_ varied greatly up to about 4.4-fold. Samples encapsulated in both whey protein (e.g., No. 3) and maltodextrin (e.g., No. 9) exhibited high protection. The samples’ protective activity was independent of inlet/outlet temperatures. As in the DPPH^•^ assay, the use of gelatin decreased the potency of samples encapsulated in whey protein but not in maltodextrin. Previous studies have shown that OMWW extracts inhibited ROS-induced DNA damage using pure DNA or cells [26,27]. However, to the best of our knowledge, this is the first study reporting the protective effect of an encapsulated OMMW polyphenolic extract against DNA damage caused by free radicals.

Based on the RACI value that each sample had in all the above assays (i.e., DPPH^•^, ABTS^•+^, ROO-induced DNA damage, and reducing power), the two most potent samples were selected in order for their antioxidant activity to be examined in endothelial cells at non-cytotoxic concentrations. These two samples were No. 3 encapsulated in whey protein and No. 9 encapsulated in maltodextrin. For assessing the samples’ antioxidant activity, GSH and ROS levels were evaluated by flow cytometry in endothelial cells. Cell treatment with both encapsulated samples showed significantly increased GSH levels, one of the most important antioxidant molecules [28]. The OMWW sample-induced increase in GSH may be due to the rescue of GSH from reaction with ROS by their direct scavenging, since OMWW encapsulated samples were shown to possess free-radical scavenging activity. Moreover, the observed increase in GSH may be attributed to increase in activity of enzymes involved in GSH synthesis and metabolism. For example, polyphenols found in olive oil have been reported to increase the expression and/or activity of glutathione peroxidase (GPx) and glutathione reductase (GR) [29]. The expression of such enzymes is mainly regulated by the transcription factor nuclear factor (erythroid-derived 2)-like2 (Nrf2) [30]. Hydroxytyrosol, one of the polyphenols identified in our OMWW extract used for the encapsulation [11], has been reported to activate Nrf2 in mouse heart [31]. Interestingly, we have also demonstrated that administration of feed containing polyphenolic extract from OMWW increased GSH in different tissues including the heart of farm animals [3,4]. Moreover, ROS levels were decreased in endothelial cells after treatment with encapsulated OMWW samples. This decrease in ROS was in agreement with the OMWW sample-induced increase in antioxidant mechanisms such as GSH in the endothelial cells as well as with OMWW samples’ free-radical scavenging activity. However, this ROS decrease was observed only in cells treated with sample No. 9 encapsulated in maltodextrin. The absence of ROS decrease in cells treated with whey protein sample No. 3 may be explained by the fact that samples’ antioxidant effects were examined in naïve cells, that is, cells that were not treated with an oxidative agent. Thus, in such cells, the baseline ROS levels are low. Moreover, sample No. 9 exhibited higher potency in free-radical scavenging assays than sample No. 3. Overall, the above findings suggested the use of OMWW polyphenolic extract encapsulated either in maltodextrin or whey protein for the development of food supplements or biofunctional foods possessing antioxidant activity, especially protection from oxidative stress-induced pathologies associated with the cardiovascular system. Interestingly, OMWW polyphenolic extract has been reported to decrease cholesterol levels in rats [32].

## 5. Conclusions

In few previous studies, maltodextrin and whey protein have been used for the encapsulation of OMWW polyphenolic extracts [21,22,23,24]. However, in the present study, there was for the first time a direct comparison between the two materials for their efficiency for the production of antioxidant powder. The results showed that both maltodextrin and whey protein were almost equally effective for the production of antioxidant powder, although in slightly different encapsulation conditions. For example, the findings indicated that, in general, inlet/outlet temperature did not affect maltodextrin samples’ antioxidant activity, but whey protein samples exhibited better antioxidant activity at lower temperatures (within the temperature range used; 100–160 °C). Gelatin was also first used in the mixture for OMWW extract’s encapsulation, but the results showed that it decreased antioxidant activity, especially in whey protein samples. Furthermore, the present study is the first demonstrating that encapsulated OMWW extract protected from ROS-induced DNA damage. Finally, it is the first time, that an encapsulated OMWW extract (as well as in general an OMWW extract) has been shown to enhance antioxidant mechanisms (i.e., GSH) in endothelial cells. Of course, further studies are needed for the elucidation of mechanisms accounting for these possible beneficial effects.

## Figures and Tables

**Table 1:** The tested powders and their encapsulation conditions.

Sample	Inlet Temperature (°C)	Outlet Temperature (°C)	Aspirator (%)	Pump (%)	MEDOLIVA (mL)	Tween80 (mL)	Whey Protein (gr)	Maltodextrin (gr)	Gelatin (gr)	H_2_O (mL)	Total Volume (mL)
1	100	66	100	5	37.5	12.5	25	-	-	175	250
2	100	72	100	5	37.5	12.5	25	-	-	175	250
3	120	82	100	5	37.5	12.5	25	-	-	175	250
4	140	90	100	5	37.5	12.5	25	-	-	175	250
5	160	98	100	5	37.5	12.5	25	-	-	175	250
6	120	71	100	5	37.5	-	25	-	5	175	242.5
7	140	79	100	5	37.5	-	25	-	5	175	242.5
8	160	96	100	5	37.5	-	25	-	5	175	242.5
9	100	57	100	10	37.5	12.5	-	25	-	175	250
10	100	67	100	5	37.5	12.5	-	25	-	175	250
11	120	67	100	10	37.5	12.5	-	25	-	175	250
12	120	76	100	5	37.5	12.5	-	25	-	175	250
13	140	84	100	5	37.5	12.5	-	25	-	175	250
14	160	86	100	10	37.5	12.5	-	25	-	175	250
15	160	96	100	5	37.5	12.5	-	25	-	175	250
16	100	61	100	5	37.5	-	-	25	5	175	242.5
17	140	80	100	5	37.5	-	-	25	5	175	242.5

**Table 2:** Free-radical scavenging activity against DPPH^•^ and ABTS^•+^ radicals, protective activity against peroxyl radical (ROO)-induced DNA damage, and reducing power of the powders.

Sample	ABTS ^a^ IC_50_ (μg/mL)	DPPH ^a^ IC_50_ (μg/mL)	ROO ^b^ IC_50_ (μg/mL)	Reducing power ^a^ 0.5 AU	RACI ^c^
1	428 ± 26 *	295 ± 24 *	800 ± 88 *	660 ± 33 *	−0.76
2	355 ± 14 *	340 ± 41 *	1580 ± 63 *	707 ± 28 *	−0.41
3	450 ± 36 *	352 ± 46 *	520 ± 41 *	434 ± 30 *	−1.13
4	500 ± 45 *	320 ± 10 *	935 ± 84 *	890 ± 18 *	−0.06
5	547 ± 16 *	330 ± 26 *	960 ± 57 *	700 ± 21 *	−0.27
6	300 ± 22 *	390 ± 16 *	725 ± 67 *	ND	−1.03
7	510 ± 44 *	385 ± 19 *	1190 ± 111 *	ND	−0.13
8	710 ± 68 *	409 ± 37 *	2300 ± 227 *	ND	1.19
9	290 ± 9 *	295 ± 18 *	590 ± 65 *	689 ± 55 *	−1.11
10	390 ± 39 *	526 ± 42 *	2075 ± 103 *	866 ± 30 *	0.55
11	590 ± 51 *	465 ± 47 *	1600 ± 149 *	ND	0.56
12	530 ± 46 *	638 ± 51 *	1250 ± 113 *	ND	0.64
13	540 ± 38 *	540 ± 16 *	2250 ± 180 *	747 ± 67 *	0.78
14	520 ± 48 *	650 ± 59 *	1200 ± 113 *	ND	0.61
15	430 ± 17 *	660 ± 73 *	1515 ± 136 *	825 ± 8 *	0.59
16	460 ± 42 *	465 ± 65 *	820 ± 77 *	ND	−0.29
17	590 ± 48 *	510 ± 25 *	1900 ± 86 *	ND	0.86
Vitamin C	4 ± 0.3 *	5 ± 0.4 *	69 ± 5 *	3 ± 0.4 *	-

^a^ Values are the mean ± SD of at least two separate triplicate experiments. ^b^ Values are the mean ± SD from three independent experiments. * *p* < 0.05, indicates significant difference from the control values. ^c^ RACI: Relative antioxidant capacity index. ND: Non-detectable at the tested concentrations.
